# The influence of postsynaptic structure on missing quanta at the *Drosophila* neuromuscular junction

**DOI:** 10.1186/s12868-016-0290-7

**Published:** 2016-07-26

**Authors:** Christine T. Nguyen, Bryan A. Stewart

**Affiliations:** 1 Department of Biology, University of Toronto Mississauga, Mississauga, Canada; 2 Department of Cell and Systems Biology, University of Toronto, Toronto, Canada

**Keywords:** Synaptic transmission, Postsynaptic, Quanta, *Drosophila*

## Abstract

**Background:**

Synaptic transmission requires both pre- and post-synaptic elements for neural communication. The postsynaptic structure contributes to the ability of synaptic currents to induce voltage changes in postsynaptic cells. At the *Drosophila* neuromuscular junction (NMJ), the postsynaptic structure, known as the subsynaptic reticulum (SSR), consists of elaborate membrane folds that link the synaptic contacts to the muscle, but its role in synaptic physiology is poorly understood.

**Results:**

In this study, we investigate the role of the SSR with simultaneous intra- and extra-cellular recordings that allow us to identify the origin of spontaneously occurring synaptic events. We compare data from Type 1b and 1s synaptic boutons, which have naturally occurring variations of the SSR, as well as from genetic mutants that up or down-regulate SSR complexity. We observed that some synaptic currents do not result in postsynaptic voltage changes, events we called ‘missing quanta’. The frequency of missing quanta is positively correlated with SSR complexity in both natural and genetically-induced variants. Rise-time and amplitude data suggest that passive membrane properties contribute to the observed differences in synaptic effectiveness.

**Conclusion:**

We conclude that electrotonic decay within the postsynaptic structure contributes to the phenomenon of missing quanta. Further studies directed at understanding the role of the SSR in synaptic transmission and the potential for regulating ‘missing quanta’ will yield important information about synaptic transmission at the *Drosophila* NMJ.

## Background

Synaptic transmission is the process used by neurons to communicate with other cells. Key to this process is the synapse, which is the very close apposition of cellular membranes of the presynaptic and postsynaptic cells. At the *Drosophila* NMJ, the neurotransmitter glutamate is secreted and binds to glutamate receptors found on the postsynaptic cell [13, 14]. The flow of cations into the postsynaptic muscle induces a voltage change activating the muscle. Surrounding the boutons of the *Drosophila* NMJ are dense postsynaptic muscle membrane folds called the subsynaptic reticulum (SSR) [3, 12, 28]. From ultrastructural analysis of the *Drosophila* NMJ, the SSR appears to be densely packed convoluted postsynaptic “leaflets” of muscle membrane, forming compartments that underlie and surround the bulbous endings of the presynaptic nerve terminal [3, 19].

Studies of the SSR have given rise to suggestions about its role in synaptic development and physiology. Some of the functions include acting as a site where glutamate receptors (GluR) are translated [36], a hub where important proteins needed for the synapse concentrate [1], and there are indications that a change in the SSR’s morphology could affect synaptic efficacy [19].

The SSR bears some similarity to junctional folds of mice, frogs and other vertebrate neuromuscular junctions. In some ways, the SSR also closely resembles it to dendritic spines typical of CNS neurons [34], which accept electrical input for synaptic function [27]. The SSR can be thought of as an electric circuit that depolarizes via the acceptance of quantal current [43].

Relatively little is understood regarding the role that the *Drosophila* SSR plays in the physiology of synaptic transmission, however some models have been proposed by previous studies, including the idea that the SSRs compartmentalization and postsynaptic micro-domains house signals that shape synaptic response [41]. It is generally assumed that when neurotransmitters activate their receptors on the postsynaptic cell, there will be a physiological response in the receiving cell. Here we report the presence of ‘missing quanta’ within the *Drosophila* larval NMJ system.

## Results

### Missing quanta

**Fig. 1:**
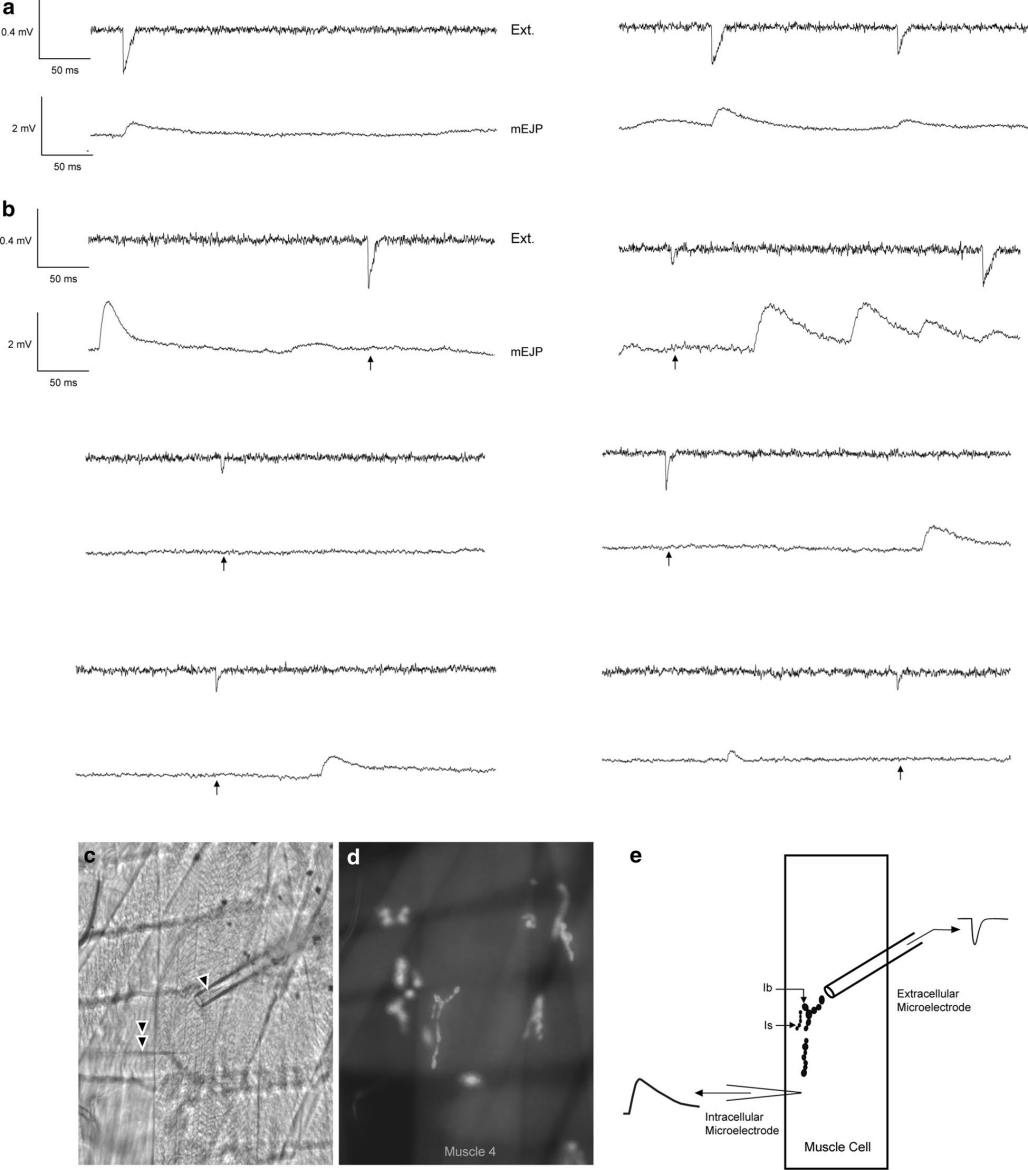
Simultaneous extracellular and intracellular recordings. **a** Examples of extracellular (Ext) recordings in which transmitter release produces a corresponding postsynaptic response detected by the intracellular (Int) electrode. **b** Example traces with missing events. An extracellular trace is shown where there is a signal detected (Ext), but with the absence of an intracellular (Int) response (no mEJP) in the intracellular trace. *Arrows* indicate the presence of an extracellular event, but a missing intracellular event. **c** Muscle 4 of *MHC::mCD8*-*GFP*-*Sh* larvae under transmitted light microscope showing extracellular (single arrowhead) and intracellular microelectrode (double arrowhead) placement. **d** Same NMJ showing the fluorescence of CD8-Sh-GFP and how boutons were selected. **e** Cartoon representation of muscle, bouton and electrode placement

The primary observation of this report is that, in our model synaptic system, not all synaptic currents result in a change in membrane voltage. We were able to make this observation by combining focal extracellular recordings with simultaneous intracellular measurements [18] (Fig. 1). The extracellular records allow us to detect synaptic events arising within the lumen of the pipette and infer the activation of postsynaptic receptors and ionic current flow. In general, about 90–95 % of the observed spontaneously occurring extracellular synaptic events resulted in a corresponding, time-locked, excitatory postsynaptic potential detected with the intracellular microelectrode. However, about 5–10 % of observed extracellular synaptic events seemingly fail to cause a corresponding change in membrane voltage. In other words, the quantal events detected with the extracellular electrode go missing.

To measure the occurrence of missing quanta we first used two control strains: *Ore*-*R* was selected to serve as a wild-type reference, whereas *w*; *MHC::mCD8*-*GFP*-*Sh* was used for live imaging and bouton identification. Synaptic boutons can be visualized with transmitted light and Differential Interference Contrast microscopy and allow for the placement of electrodes. However, fluorescently labeled boutons are easier to identify. The *w*; *MHC::mCD8*-*GFP*-*Sh* strain expresses a chimeric protein consisting of the CD8 transmembrane domain, GFP, and the cytoplasmic tail-domain of the Shaker potassium channel, under the control of the *Myosin heavy chain* promoter [46]. Thus, CD8–GFP–Shaker is expressed in all muscles and it has been previously demonstrated that the chimeric protein accumulates in synaptic boutons and is a useful marker for live imaging and bouton identification.

We performed simultaneous recordings from 1b or 1s boutons and collected 50 extracellular events from 20 larval samples for each bouton from each genotype and thus analyzed 1000 synaptic events for each data set. A missing event was tallied when an extracellular synaptic event was detected with an extracellular electrode, however no corresponding intracellular response is detected from the intracellular electrode. As shown in Table 1, we found a higher percentage of missing events for Type 1b compared to Type 1s boutons.

**Table 1:** Comparison of the percentage of missing events for Type 1b and 1s boutons of control genotypes

Bouton type	Missing events (% ±SEM)	
	1b	1s
*Oregon*-*R*	7.7 ± 0.6	3.5 ± 0.4
*MHC::mCD8*-*GFP*-*Sh*	6.2 ± 0.5	1.8 ± 0.3

1000 extracellular spontaneous events (50 events from 20 boutons) were collected from each genotype and bouton type, and scored for whether a corresponding intracellular mEJP occurred

Having shown that there are missing quanta at larval *Drosophila* neuromuscular synapse and that there are differences between Type 1b and 1s boutons, with a greater preponderance of missing events at Type 1b boutons, we considered what may give rise to missing quanta.

While there may be several molecular and/or biochemical signatures that distinguish the two bouton types, for the remainder of this study we will focus on the well-known structural differences of the subsynaptic reticulum (SSR). It has been previously shown that the closely apposed presynaptic and postsynaptic membranes are connected to the muscle by the highly convoluted and tortuous membrane system of the SSR and that Type 1b boutons are composed of a larger and more complex SSR than Type 1s boutons [3]. Indeed, one the main reasons the boutons appear ‘big’ and ‘small’ in the light microscope is the extent of the SSR.

There are several possibilities that may link SSR complexity to a role in the phenomenon of missing quanta. Since the SSR is an electrical connection between the point of synaptic contact and the postsynaptic muscle cell, the SSR likely acts as an electrotonic pathway between synapse and muscle. Therefore, passive membrane properties likely play a role, akin to the dendritic connection between dendritic spines and cell body of CNS neurons. In order to investigate whether electrotonic properties influence the existence of missing quanta we employed genetic variants of *Drosophila* that either increase or decrease SSR complexity. We predicted that, as with Type 1b and 1s boutons, the proportion of missing events should vary in the same direction as SSR morphometry in genetic variants.

### Postsynaptic expression of Ral affects SSR width

Mutations of the Ral gene have been recently shown to increase or decrease the complexity of the SSR. Expression of *Ral*^*G20V*^ in muscle increases the amount of SSR, whereas the *ral*^*G0501*^ allele reduces Ral protein expression and causes a decrease in the width and complexity of the SSR [41]. We investigated the occurrence of missing quanta in these alleles.

**Fig. 2:**
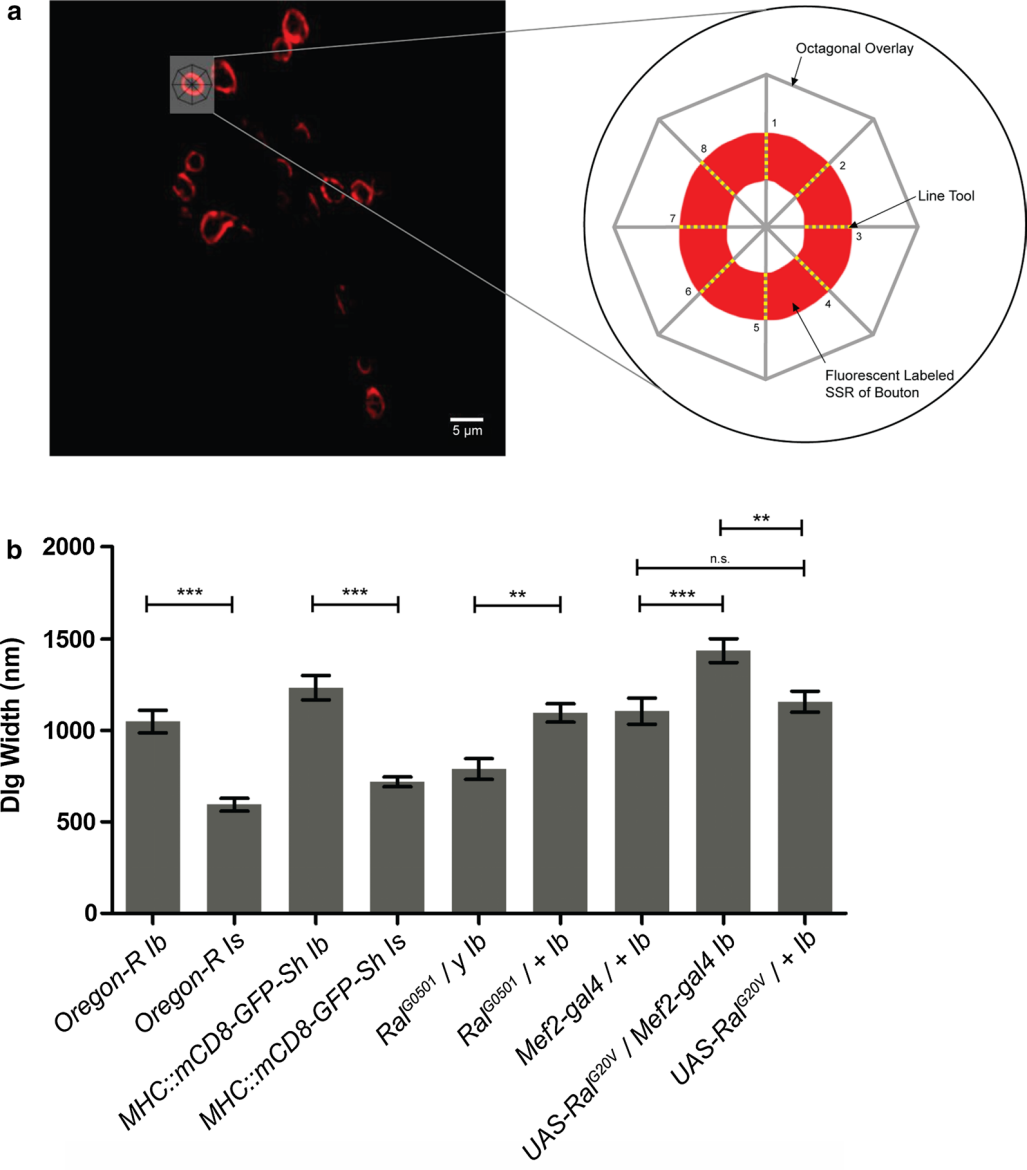
Quantification of Dlg width. **a** Anti-Dlg was used to immuno-label the SSR and images were analyzed with ImageJ software. An octagonal overlay was placed on the image and a line tool was used to quantify the average length of eight spokes, producing the average width of Dlg fluorescence for a single bouton type. **b** The quantification of Dlg width for each genotype, where each *bar* represents the mean ± SEM. *n* = 15 boutons for each genotype (One-way ANOVA Bonferroni post test, not significant *n.s.*, **p < 0.001; ***p < 0.0001)

Although previous studies have quantified the extent of the SSR in these alleles using electron microscopy [41], we sought to corroborate the previous findings (Fig. 2). We measured SSR width using anti-Dlg immunocytochemistry along with laser scanning confocal microscopy. Dlg fluorescence was used as a proxy to measure SSR width. As expected Type 1b boutons from *Ral*^*G20V*^ expression showed the largest SSR, while Type 1b boutons of *ral*^*G0501*^ mutants were smaller. The SSR width in the control strains, *OreR* and *MHC::mCD8*-*GFP*-*Sh* fell between these two extremes. Type 1s boutons of the *OreR* and *MHC::mCD8*-*GFP*-*Sh genotype* contains the smallest SSR widths of 0.6 μm ± 0.03 and 0.7 μm ± 0.02 respectively (mean ± SEM). All other genotypes *OreR* Type 1b (1.02 μm ± 0.05), *MHC::mCD8*-*GFP*-*Sh* Type 1b (1.1 μm ± 0.06), and genotypes reared in a control background *Mef2*-*gal4/*+ (1.1 μm ± 0.05)*, ral*^*G0501*^*/*+ (1.1 μm ± 0.04), and *Ral*^*G20V*^*/*+ (1.1 μm ± 0.05) had varying similar SSR widths. 15 Type 1b boutons for each genotype and 15 Type 1s boutons for *OreR* and *MHC::mCD8*-*GFP*-*Sh* larvae was measured (*n* = 15).

### Measuring mEJP amplitudes

**Fig. 3:**
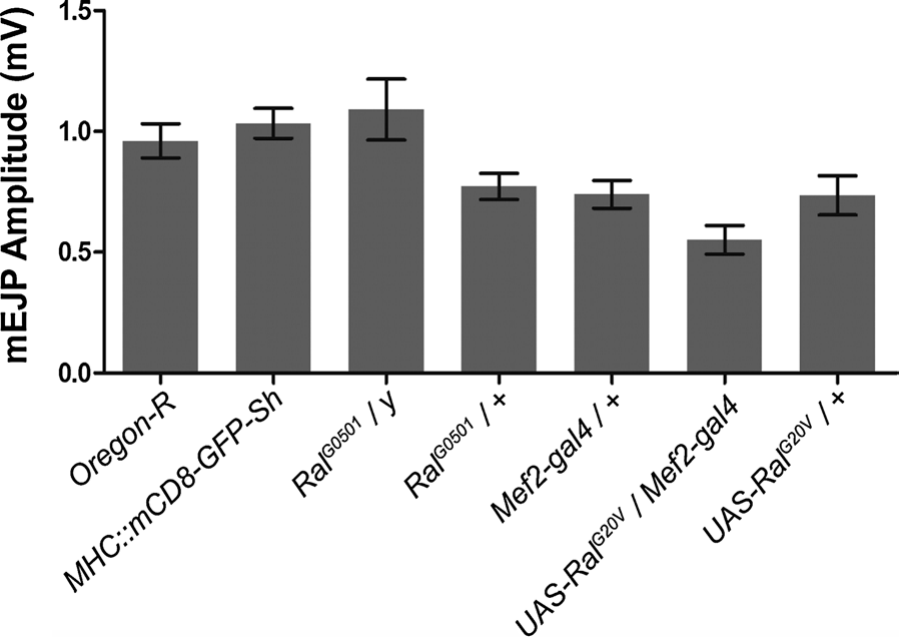
Electrophysiological analysis of spontaneous miniature potential (mEJP) amplitudes of all genotypes. mEJPs were recorded *en masse* from muscle abdominal segments A3–A5 of muscle 4 for all genotypes, except for genotypes *Ral* ^*G0501*^ */y* and *Ral* ^*G0501*^ */*+, where recordings were taken from muscle 6. Data is presented as the mean ± SEM amplitude of 50 mEJP for 10 larvae of each genotype (*n* = 10) (One-way ANOVA Bonferroni post test, there are no significant differences between groups)

Before in-depth analysis of missing quanta, we collected mEJPs *en masse* from the test and control genotypes in the usual manner, analyzing 50 mEJPs from 10 muscle cells per genotype (*n* = 10) (Fig. 3). Data were recorded from muscle 4 of abdominal segments A3–A5 for *OreR*, *MHC::mCD8*-*GFP*-*Sh, Mef2*-*gal4/OreR, UAS*-*Ral*^*G20V*^*/*+, *UAS*-*Ral*^*G20V*^*/Mef2*-*Gal4,* larval genotypes (Fig. 3). Muscle recordings for *ral*^*G0501*^*/Y*, and *ral*^*G0501*^*/*+ larvae were done on muscle 6 due to the difficulty of obtaining a resting membrane potential less than −60 mV for this genotype in muscle 4. In addition, the muscle membrane input resistance also measured and ranged between 5 and 10 MΩ. Intracellular mEJPs were recorded to investigate any amplitude variations between mutant and control larvae.

The amplitude of mEJPs from control *OreR* and *MHC::mCD8*-*GFP*-*Sh* larvae showed similar results, with a range of mean amplitudes of 0.9–1 mV. All other genotypes showed similar amplitudes, with no significant differences in a range of 0.6–0.9 mV (*p* > 0.05, One-way ANOVA).

### Missing quanta in larvae with expanded or reduced SSR

**Fig. 4:**
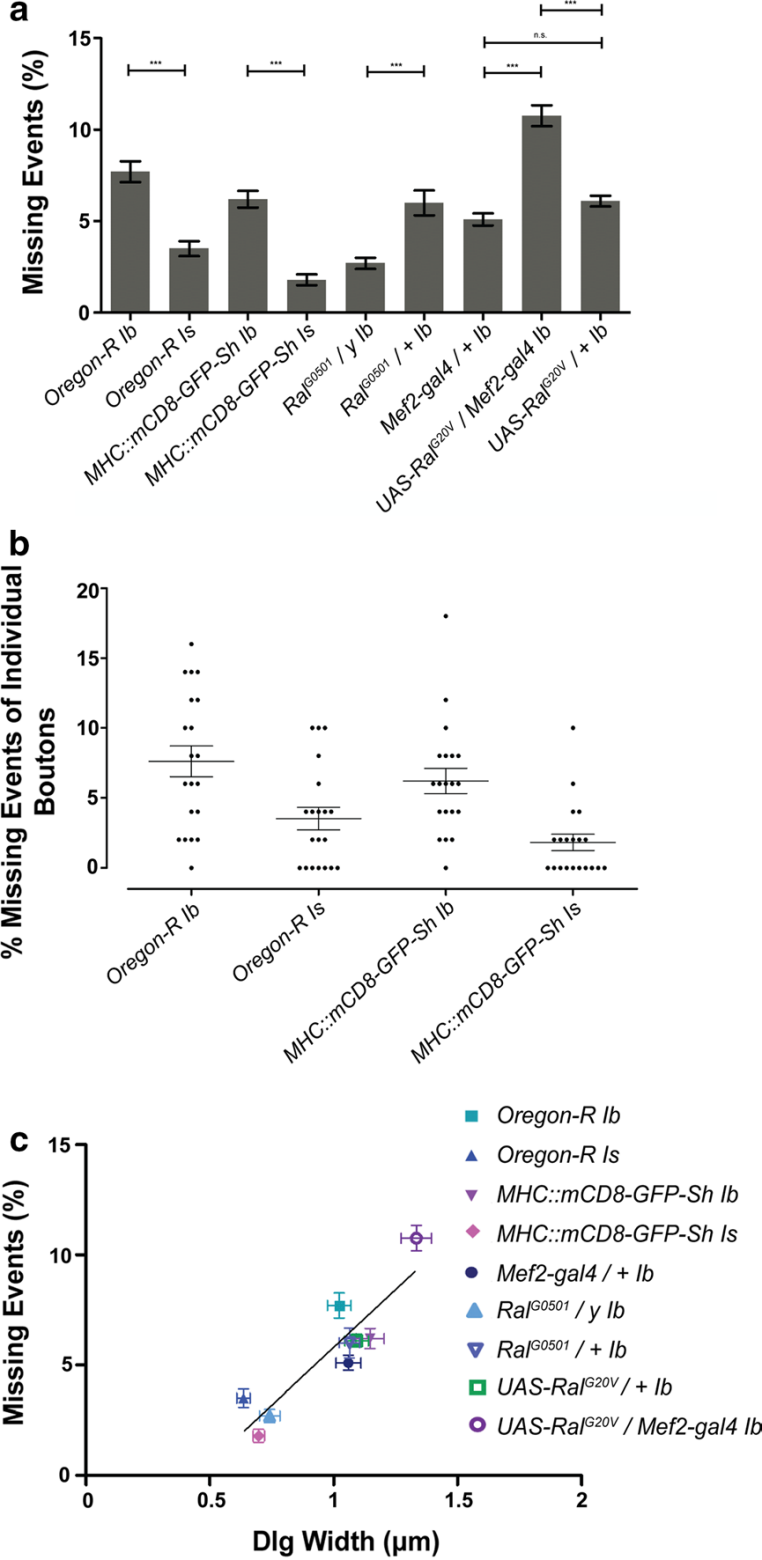
The relationship between missing events and SSR complexity. **a** The proportion of missing quanta from indicated genotype and bouton type. *Each bar* represents the mean ± SEM missing intracellular spontaneous miniature potential (mEJP) as a percentage. 50 extracellular events recorded from 20 Type 1b boutons of each genotype (*n* = 20), as well as 50 extracellular events recorded from 20 Type 1s boutons of of the *OreR* and *MHC::mCD8*-*GFP*-*Sh* genotype (*n* = 20), for a total of 1000 events analyzed per data point (One-way ANOVA, Bonferroni post tests for relevant genetic comparisons, not significant *n.s.*, ***p < 0.0001). **b** The percent of missing events for individual bouton recordings of Type 1b and Type 1s *OreR* and *MHC::mCD8*-*GFP*-*Sh* larvae. Each point represents the percent of missing events for an individual bouton analyzed from 50 intracellular events of 20 boutons for each genotype (*n* = 20). *Error bars* represent the mean ± SEM (One-way ANOVA, Bonferroni post test, **p < 0.001). **c** The relationship between percent missing events and SSR size is shown in where each point represents the mean ± SEM of percent missing events and SSR complexity (using Dlg width (μm)). The linear regression line shows a positive slope of 10.4 % missing events per micron Dlg width and with an r^2^ value of 0.8. The slope is statistically different from zero

As described above for the control strains, we next quantified the extent of missing quanta in the larvae with expanded or reduced SSR. Interestingly, we found that genotypes with expanded SSR showed a greater proportion of missing events than those with reduced SSR (Fig. 4a). *UAS*-*Ral*^*G20V*^*/Mef2*-*gal4* contains the highest mean proportion of missing intracellular events, showing 10.8 % missing events. In contrast, *ral*^*G0501*^, which had the smallest Type 1b SSR, also showed the fewest proportion of missing intracellular events, with a mean value of 2.7 %.

To further analyze these data, and help us determine if there is a general relationship between SSR and missing quanta, we plotted SSR width versus missing events, for all bouton types and genotypes (Fig. 4b). This analysis showed us that there is a positive relationship between these two parameters: as SSR width increases so does the proportion of missing quantal events. Linear regression of these data shows that there is a positive slope of 10.4 % of missing events per µm Dlg width and an r^2^ value of 0.8. For example, the Type 1s boutons from control strains and Type 1b from *ral*^*G0501*^*/y* have the smallest mean SSR widths and also the fewest proportion of missing events, while *UAS*-*Ral*^*G20V*^*/Mef2*-*gal4* have the largest SSRs and also the greatest proportion of missing quanta.

### 20–80 % rise times of identified mEJPs

**Fig. 5:**
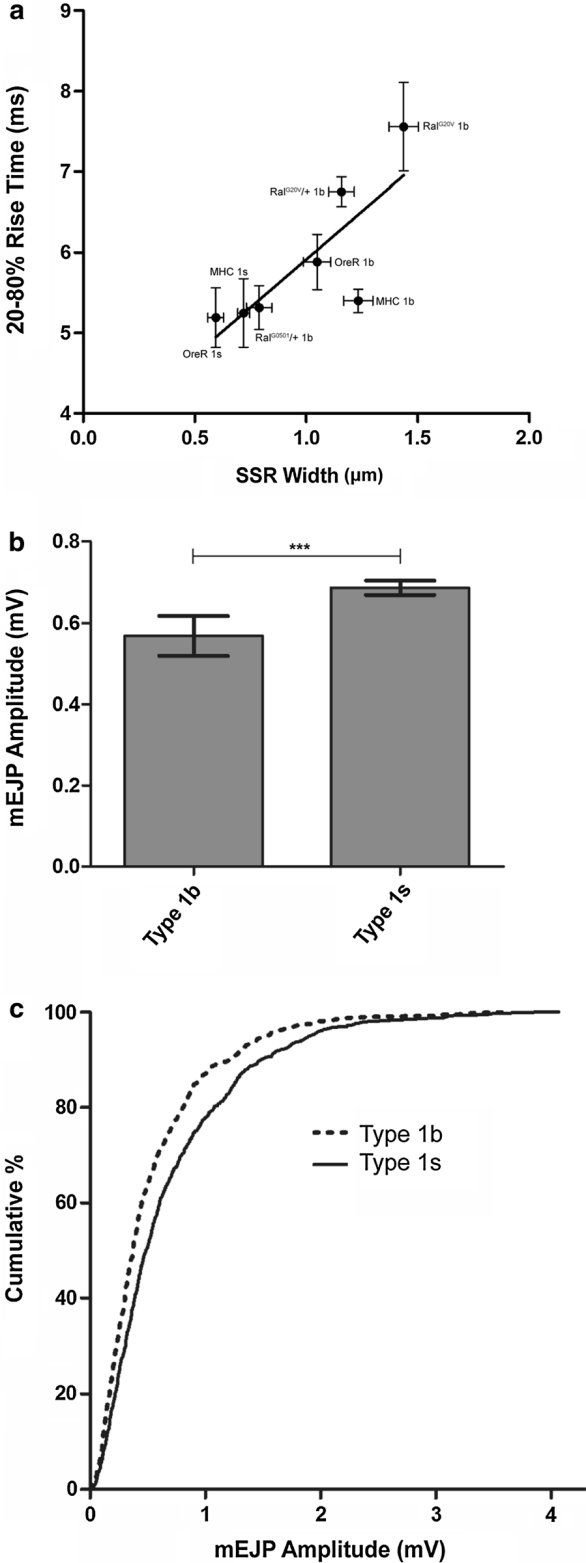
Influence of passive membrane properties on mEJPs. **a** The 20–80 % rise time of identified mEJPs was analyzed from each of the indicated genotypes and plotted against the measured SSR width. The linear regression line shows a positive slope of 2.4 ms/µm Dlg width with an r^2^ of 0.64. The slope is statistically different from zero. **b** The amplitude of mEJPs collected from identified *OreR* Type 1b (973 mEJPs) and Type 1s (935 mEJPs) boutons were analyzed. The *bars* represent the mean ± SEM amplitude for each bouton type, with a significant difference between the two (*Mann*–*Whitney test*, *p* < 0.0001). **c** Cumulative frequency histogram showing the distribution of mEJP amplitudes for each bouton type. mEJPs from Type Ib boutons are more frequently smaller than from Type 1s

The above data support the idea that SSR complexity is correlated with the extent of missing quanta. If the SSR is indeed acting as an electronic connector between synapse and muscle, further predictions can be made. For example, due to the passive membrane properties of the SSR we would expect to see effects on the time-dependent characteristics of the mEJPs. Since we collected simultaneous intra- and extra-cellular recordings from identified boutons and can identify the origins of a subset of mEJPs, we analyzed the 20–80 % rise-time of identified mEJPs as a parameter sensitive to changes in electrotonic path length. We found that the greatest 20–80 % mEJP rise-times occurred in *UAS*-*Ral*^*G20V*^*/Mef2*-*Gal4* larvae, with a rise-time of 7.55 ± 0.5 ms, whereas the mEJPs from *OreR* Type 1s boutons had the shortest rise times of 5.19 ± 0.36 ms. Other bouton types fell in between these two values as plotted in a regression versus SSR width (Fig. 5a). The slope of the regression line is 2.3 ms/µm, an r^2^ value of 0.6, and the slope is significantly different from zero.

We also attempted to measure the mEJP decay time constant. However, because it is a much slower process than rise time, it is contaminated by the prevailing background of low frequency changes in the muscle membrane potential not related to mEJPs. Without a more favorable signal:noise ratio we could not make definitive conclusions about this parameter.

Lastly, we analyzed the amplitude measured of the identified mEJPs. Cable theory also predicts an effect of electronic path length on mEJP amplitude and so we measured the amplitude of 973 mEJPs from *OreR* Type 1s boutons and 935 mEJPs from *OreR* Type 1b boutons. We found that on average the mEJPs from Type 1s boutons were 0.68 ± 0.02 mV, whereas mEJPs were 0.56 ± 0.04 mV (Fig. 5b) from Type 1b boutons (*p* < 0.0001, Bonferroni post test). A frequency distribution revealed a greater proportion of smaller mEJPs recorded from the Type 1b boutons (Fig. 5c).

## Discussion

Here we report the presence of missing quanta at the *Drosophila* neuromuscular synapse. We provide evidence that the proportion of missing quanta is positively correlated with the extent of the SSR. The most powerful support for this idea comes from the naturally occurring variation in SSR complexity between the Type 1b and Type 1s boutons in the control strains. Because the genetic background of these strains is essentially wild-type, without genetic perturbations to induce changes in SSR complexity and the two bouton types occur on the same postsynaptic target, we conclude that missing quanta are part of the natural physiology of this synapse. The relationship between SSR width and missing quanta was borne out when strains known to expand or reduce the SSR were examined and the proportion of missing quanta co-varied in the same direction.

### Origins of missing quanta

Our data lead us to propose that an electrotonic connection between synapse and muscle significantly modulates the electrical response of the muscle cell. Accordingly, the passive membrane properties of the SSR likely cause the decay of excitatory potentials along its path. The simplest explanation consistent with our data is that electrotonic decay of synaptic potential along the SSR results in some synaptic currents failing to induce changes in muscle membrane voltage, measured by the intracellular electrode. Cable theory predicts that mEJP amplitude should also be smaller with the longer pathway, which we also demonstrate by showing that the mEJP amplitude from identified 1b boutons is smaller than from 1s boutons. Finally, consistent with our suggestion, we measured the 20–80 % rise time of identified mEJPS and found that there was also a positive correlation between SSR width and mEJP rise time.

It is interesting to note that the mEJP amplitudes reported in Fig. 5 are slightly smaller than those in Fig. 3. We attribute the variance to differences in the recording configuration. With *en masse* intracellular recordings shown in in Fig. 3, mEJPs are recorded from potentially ~50 of Ib and Is boutons on the muscle, potentially each contributing different mEJP sizes. With identified boutons shown in Fig. 5, we are sampling from only 1 recording site per muscle.

Interpretation of our results is also complex because of the highly variable nature of some of the parameters known to distinguish Type 1b and 1s boutons. We know, for example, that both bouton-types generate synaptic currents with an amplitude distribution severely skewed towards smaller events (as indicated in Fig. 5c, for example). Karunanithi et al. [16] also showed that spontaneously occurring quantal currents from Type 1s boutons, on average, are larger than those from Type 1b boutons. They attributed some of this variation to a larger vesicle diameter in the Type 1s terminal, compared to 1b terminals, although whether volume is the key parameter is not clear [45]. Here we corroborate that mEJPs associated with the two terminal types are distinct: mEJPs from 1b boutons are on average smaller than those from 1s boutons. However, in light of the present data it seems that the differential complexity of the SSR is also a contributing factor.

The SSR is a heterogeneous structure. Even casual inspection of electron micrographs reveals that the SSR is a very dense and convoluted membrane system. We can imagine that particularly thin SSR connections could have a sufficient longitudinal resistance to essentially nullify the effect of synaptic currents. In this respect the SSR acts like a dendrite of CNS neurons dendrites [31, 35]. Dendritic morphology is known to have a significant impact on postsynaptic potentials measured at the cell body, influencing both amplitude and time course of synaptic events (reviewed by Magee [23]).

Our results are also informed by prior work on the postsynaptic folds found at vertebrate neuromuscular junctions. For example, frog, mouse, rat, snake and human neuromuscular synapses all have postsynaptic folds [5, 11, 25, 39, 44], with varying degrees of elaboration [38]. The prevailing model of their function is that the enrichment of voltage-gated sodium channels in the troughs of the fold act as an amplifier to ensure that synaptic depolarizations result in muscle fiber action potentials [24, 43]. Synapses that tend to release more quantal units per impulse, such as frogs, tend to have less elaborate folds whereas synapses that release fewer quantal units, such as humans, have more elaborate folds [38, 43]. This correlation, along with the enrichment of voltage-gated sodium channels in the troughs of the folds, is likely related to the safety factor required to ensure membrane depolarization of twitch muscle fibers [44].

A feature that distinguishes vertebrate and *Drosophila* larval muscle systems is that the larval muscles lack voltage-gated sodium channels and work through graded contractions. *Drosophila* larval muscles do have voltage-gated calcium channels [9, 30], but it is thought that these muscles generally do not normally generate calcium-dependent action potentials; it is not known if such channels accumulate in specific sub-regions of the SSR. Further, it is also not presently clear whether voltage-gated calcium channels are required to activate muscle contraction because calcium can also enter the muscle through the synaptic glutamate receptors [7]. Without evidence for aggregation of inwardly conducting voltage-gated cation channels, it seems less likely the *Drosophila* SSR acts as an amplifier, but rather since these nerve terminals are high-output terminals, the SSR may act as a filter to dampen the synaptic response.

There are at least two alternative hypotheses to explain missing quanta that we cannot rule out at the present time, but which seem less likely. Firstly, it is possible that there are voltage-gated ion channels in the SSR that short-circuit or shunt synaptic currents. There are no known inhibitory ligand-gated channels in this system, but there are postsynaptic Shaker K^+^-channels and slowpoke calcium-activated K^+^-channels [2, 15, 37]. However, the activating-voltage for such channels is substantially more positive than the resting membrane voltage [20] and it seems unlikely that such channels would be activated by spontaneous synaptic currents, since they usually depolarize the membrane by less than 1 mV (at the microelectrode). Secondly, we can imagine that fission/fusion machinery may break or recruit SSR synaptic plateaus and their connection to the muscle. We have no information to support or refute this possibility, but the presence of proteins in the SSR such as exocyst components like Sec5 [41], as well as BAR domain proteins, such as Amphiphysin and Syndapin [17, 21, 32] suggest dynamic regulation of SSR membranes is a possibility.

### Comparative physiology of synaptic strength

The dual motor innervation of *Drosophila* larval muscle provides the opportunity to study the mechanisms that regulate differential synaptic strength of identified neurons that innervate the same target. This situation is similar to that of the tonic and phasic innervation pattern of some crustacean neuromuscular systems, which have been studied in detail [4, 26]. Though the Type 1b boutons are bigger, they produce a smaller EJP than Type 1s boutons and Type 1b synaptic transmission shows greater high-frequency facilitation than Type 1s [18, 22]. Most studies to date have focused on presynaptic mechanisms. For example, differences in presynaptic calcium handling have been explored [10, 16], as well as the contribution of presynaptic mitochondria [8]. As noted above, ultrastructural investigations have reported that vesicle size is another feature of differentiation between Type 1b and 1s boutons [16]. Here we add that the known postsynaptic differences in the SSR morphology also likely have a functional impact on the observed differences in synaptic transmission between Type 1s and Type 1b boutons.

Our data also suggest careful attention needs to be paid to the method by which quantal content estimates are calculated. To determine quantal output, most studies, including our own previous work, divide the mean amplitude of a compound EJP (i.e. both axons are stimulated) by the average mEJP amplitude, which was measured from the ensemble of mEJPs recorded from the whole muscle. The present results show that the quantal release of a nerve terminal would be underestimated by such a method. This calculation would be particularly effected by genetic variants that impact SSR structure, since as SSR complexity increases we might expect a greater number of missing quanta and therefore a greater error in estimates of quantal content. Our corroboration that mEJPs arising from the different nerve terminals have different amplitudes suggest that quantal size also contributes to the variation of EJP size, with Type 1b boutons having smaller mEJPs on average. Thus any genetic variants that tend to alter the relative proportion of mEJPs arising from one or the other bouton type would have a differential impact on estimates of average mEJP amplitude and therefore quantal content.

Altogether our data introduce a new physiological parameter that may be a contributing determinant of synaptic strength. Further studies aimed at understanding whether the proportion of missing quanta is a physiological parameter that can be dynamically regulated will yield further understanding of the way in which postsynaptic structure influences synaptic transmission.

## Conclusion

In summary, we have demonstrated that not all synaptic currents generate postsynaptic voltage changes at the *Drosophila* larval neuromuscular junction. We call these events missing quanta and they occur at a rate of 3 % of Type 1s and 8 % of Type 1b of synaptic events in the wild-type *OreR* larvae. We observed a positive correlation between SSR width and the proportion of missing quanta, in both the natural variants and by using alleles of the *Ral* gene which can up- and down-regulate SSR complexity. In turn, we hypothesized that SSR membranes act as an electronic connection between the synapse and muscle and present evidence that mEJP rise-time and amplitude vary in a manner consistent with this hypothesis. Further studies aimed at understanding whether the phenomenon of missing quanta can be dynamically regulated will prove fruitful to understanding the role of postsynaptic structures in synaptic effectiveness.

## Methods

### Fly stocks and strains

All flies were maintained at 23–25 °C on Bloomington standard fly food medium. In this study the wild-type strain used was Oregon-R (*OreR*). The following stocks used were: *MHC::mCD8*-*GFP*-*Sh*, *UAS*-*Ral*^*G20V*^*, Ral*^*G0501*^, which were all gifts from the lab of Dr. Thomas Schwarz. *OreR* and *MHC::mCD8*-*GFP*-*Sh* larvae served as a control for this study. Control fly strains used were *Mef2*-*gal4/*+, *ral*^*G0501*^*/*+, and *UAS*-*ral*^*G20V*^*/*+. *UAS*-*ral*^*G20V*^ were expressed in the muscle by crossing these stocks to *Mef2*-*Gal4* in the UAS-Gal4 system [6].

Fly strains having a reduction in the SSR used were from the cross of *Ral*^*G0501*^*/FM7*, *Actin::GFP* with *OreR*. Only male progeny *ral*^*G0501*^*/y* were experimental since the expression of the Ral^G0501^ gene belongs on the first chromosome. Individual controls for each experimental line were also generated by crossing *UAS*-*Ral*^*G20V*^ and *UAS*-*ral*^*G0501*^*/FM7, Actin::GFP* with *OreR*. All *UAS*-*Ral*^*G20V*^*/*+ and female progeny of *UAS*-*ral*^*G0501*^*/*+ were chosen for experimental controls. Mef2-gal4 flies were crossed with *OreR* and also served as an experimental control: *Mef2*-*gal4/*+. When crossing flies, 5–10 virgin females were selected and placed into a food vial along with 5 males.

#### Electrophysiology

Intracellular and extracellular recordings were performed on dissected wandering third instar larvae in HL3 bath solution [40] with added CaCl_2_ to a total concentration of 1 mM. Glass electrodes were made using a micropipette puller (Sutter Instrument). Sharp intracellular microelectrodes were pulled and backfilled with 3 M KCl; they had resistances of ~30 MΩ. Extracellular electrodes for focal recordings [18, 33, 42] were fabricated by first pulling a long sharp pipette and then manually breaking off the tip. An angle was created in the shank of the pipette and the tips were polished using a microforge (GlassWoRx). The extracellular macropatch electrode was backfilled with HL3 solution containing 1 mM CaCl_2_, and was used to place over the bouton of choice on the NMJ. The tip of the pipette was polished using the microforge to an internal diameter of 6–8 µm, large enough to cover Type 1b boutons.

Both intracellular and extracellular currents are recorded using an AxoClamp 2B amplifier (Axon Instruments). The analog signals were converted to a digital signal using a Digidata 1322A (Axon Instruments), and further analyzed using pClamp 8.2 software. Only muscles possessing an initial resting membrane potential of anything more negative than −60 mV was recorded. Intracellular and extracellular recordings were recorded simultaneously for 2–6 min. A total of 20 animals per genotype of each bouton type (Type 1b and Type 1s) was used for the electrophysiological recordings (*n* = 20).

All electrophysiology recordings were further analyzed using Clampfit 10.0 software. The amplitudes of the miniature events, both intra- and extracellular, were measured using the difference of two cursors, with one placed at the baseline of an event and the other at the peak of the event. EJPs were measured by first setting the baseline of all events (16 sweeps) to 0. Cursors were then used to measure the amplitude of the average trace. All data was then transferred to GraphPad Prism 5 for graphical and statistical analysis.

### Immunocytochemistry

Dissected larvae are fixed with 4 % paraformaldehyde (Ted Pella, Inc) in phosphate buffered saline (PBS) for 20 min. Larvae were then washed in PBS with 0.1 % Triton X-100 (PBT). Larvae are then transferred into a new microcentrifuge tube containing PBT and are then blocked in blocking solution (3 % bovine serum albumin and 2 % Normal goal serum) for 30 min. Primary antibody was then added and allowed to incubate over night at 4 °C.

Monoclonal mouse anti-Discs large 4F3 [29] was used at a dilution of 1:200 and was obtained from the Developmental Studies Hybridoma Bank at the University of Iowa. Following incubation with primary antibody, larvae were washed 3 times for 10 min each in PBT. The secondary antibody Alexa Fluor^®^ 555 (Life Technologies) was added (1:500) and allowed to incubate for 1 h at room temperature. Larvae were then washed in PBT for a final 4 times at 15 min each wash. All stained larvae are then placed on glass slides containing Vectashield mounting medium for fluorescence (Vector Labs).

Images were collected on a Zeiss LSM 510 confocal microscope. Snap shots and Z-stacks were taken from muscles 4, 6, and 7 using a 40×/1.4 oil immersion lens or a 63×/1.4 oil immersion lens.

### Quantification of SSR width

Images were analyzed on ImageJ 1.48 Software where a Z-projection was created allowing all Z-stacks to stack to a single image showing maximum intensity from all stacks, or the stack showing the optimum intensity for a specific bouton was selected for measurement. An octagonal overlay with 8 visible connected spokes is placed over the bouton to be measured. Using the ImageJ line tool, lines are drawn on the octagonal spokes only where fluorescence is present. A total of 8 lines with varying lengths are recorded for each bouton and then were averaged to obtain the average width (μm) of Dlg fluorescence for that bouton (Fig. 2a). Mean Dlg widths for this study is based on a total of 10 boutons for each genotype.

### Statistical analysis and sample size

All statistical analysis was performed on GraphPad Prism 5 software. Student *t* test, ANOVA group comparisons, and column statistics were used on the data presented. A significance level of *p* < 0.05 was used for all experiments.

## Abbreviations

NMJ: neuromuscular junction
SSR: subsynaptic reticulum
EJP: excitatory junctional potential
mEJP: miniature excitatory junctional potential
